# Specificity of the *E. coli* LysR-Type Transcriptional Regulators

**DOI:** 10.1371/journal.pone.0015189

**Published:** 2010-12-20

**Authors:** Gwendowlyn S. Knapp, James C. Hu

**Affiliations:** 1 Department of Biochemistry and Biophysics, Texas A&M University, College Station, Texas, United States of America; 2 Texas AgriLife Research, Texas A&M University, College Station, Texas, United States of America; Baylor College of Medicine, United States of America

## Abstract

**Background:**

Families of paralogous oligomeric proteins are common in biology. How the specificity of assembly evolves is a fundamental question of biology. The LysR-Type Transcriptional Regulators (LTTR) form perhaps the largest family of transcriptional regulators in bacteria. Because genomes often encode many LTTR family members, it is assumed that many distinct homooligomers are formed simultaneously in the same cell without interfering with each other's activities, suggesting specificity in the interactions. However, this assumption has not been systematically tested.

**Methodology/Principal Findings:**

A negative-dominant assay with λcI repressor fusions was used to evaluate the assembly of the LTTRs in *E. coli* K-12. Thioredoxin (Trx)-LTTR fusions were used to challenge the homooligomeric interactions of λcI-LTTR fusions. Eight cI-LTTR fusions were challenged with twenty-eight Trx fusions. LTTRs could be divided into three classes based on their interactions with other LTTRs.

**Conclusions/Significance:**

Multimerization of LTTRs in *E. coli* K-12 is mostly specific. However, under the conditions of the assay, many LTTRs interact with more than one noncognate partner. The physiological significance and physical basis for these interactions are not known.

## Introduction

The LysR-Type Transcriptional Regulators (LTTRs) are a diverse family of oligomeric bacterial transcription factors. Initially identified by Henikoff *et al*., their number has grown to over 40,000 potential members (IPR000847 HTH_LysR) as of this writing, making it perhaps the largest family of transcriptional regulators among prokaryotes [1], [2]. Characterized LTTRs regulate a wide variety of transcription units in response to a wide variety of environmental signals. In *E.coli* K-12 LTTRs regulate nitrogen source utilization, amino acid biosynthesis and catabolism, oxidative stress response and detoxification of the cell [3], [4]. BenM and CatM both affect aromatic compound degradation in Acinetobacter sp. strain ADP1 [5], [6]. RovM of *Yersinia pestis*, MvfR of *Pseudomonas aeruginosa* PA14, and AphB of *Vibrio cholerae* have all been shown to be involved in virulence [7], [8], [9], [10].

As a large family of oligomeric proteins presumably derived from a common ancestor, the LTTRs provide an excellent opportunity to investigate the evolution of protein self-assembly. Those LTTRs that have been characterized so far form homooligomers, consisting mostly of tetramers and in some cases, dimers [1], [3], [11]. Examination of the available crystal structures of full-length LTTRs and a larger number of oligomeric regulatory domains shows an overall structural similarity, with the interactions being essential for function of the protein [12], [13], [14], [15], [16], [17].

The number of LTTRs in a given species is highly variable. For example, the *E coli* K-12 strain MG1655 has 47 members, while *Pseudomonas aeruginosa* PA01 has 121 members (COG0583 [18]). By contrast, LTTRs are absent from the genome of *H. pylori*. In species with large numbers of LTTRs, it could be potentially detrimental if all combinations of LTTRs formed heteromultimers, suggesting that there is a strong selective pressure for complex formation to be highly specific. However, the specificity of the oligomerization by LTTRs has not been explicitly tested. In this study, we utilize λcI repressor LTTR fusions in a negative-dominance assay [19] to examine whether LTTRs from *E. coli* form promiscuous heterotypic interactions.

## Results

### A Negative-Dominance System with λ repressor and Thioredoxin Fusions

**Figure 1:**
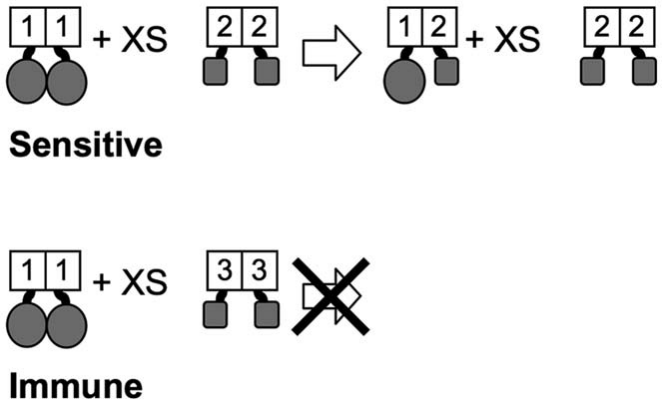
Negative-dominance Assay. The oligomerizing cI fusion is shown on the left-hand side. The proteins tested for heterotypic interactions are represented by the colored spheres. Proteins that can form heterotypic interactions with the immune cI fusion generate a sensitive phenotype. Those that can not form an interaction remain immune.

The basic idea of our assay is shown in Figure 1. A subset of the *E. coli* LTTRs are able to drive oligomerization of the λ repressor N-terminal DNA-binding domain at levels sufficient to confer immunity to infection by bacteriophage λ. Overexpression of a competing oligomerization domain that is not fused to the DNA binding domain of λ cI will render cells sensitive to λ if formation of heteromultimers reduces the intracellular level of homomultimeric repressor fusions below the amount needed to repress infecting phage.

Previous versions of a negative-dominance system with λ cI repressor fusions utilized two constructs: a λ cI repressor fusion that could homooligomerize and a λ cI repressor fusion with a mutation in the helix-turn-helix DNA binding domain that abolished binding of the fusion to DNA [19]. Instead of the latter construct, we utilized a thioredoxin (Trx) fusion that was under the control of P_araBAD_.

**Figure 2:**
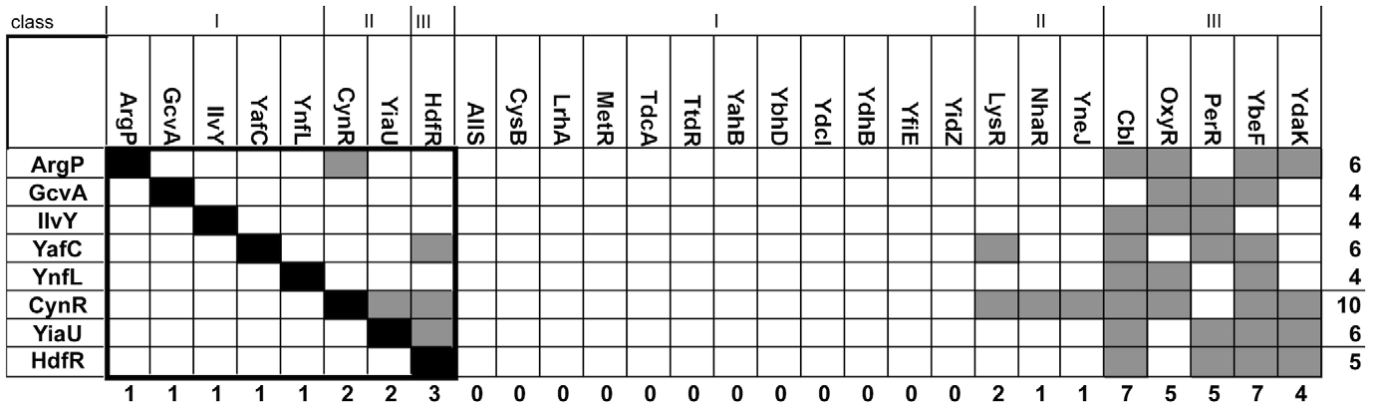
Interaction Grid of the LTTRs. On the left are the immune λ cI repressor fusions. Across the top are the Trx-LTTR fusion constructs. The type of interaction between two proteins is denoted by a shaded square. Black is representative of a homotypic interaction, while gray is a heterotypic interaction. White indicates no interaction was detected.

LTTRs with the ability to oligomerize as λ cI repressor fusions were initially identified in a large scale screen of the *E. coli* genome [20]. We challenged eight of these λ cI-LTTR repressor fusions with their Trx-LTTR fusion counterparts, using fusions to the yeast GCN4 leucine zipper domain, Trx-GCN4 and λcI-GCN4, as controls. This small set of interactions is outlined in the black box in Figure 2. The Trx-GCN4 did not interact with any repressor fusions except λ cI-GCN4. λ cI-GCN4 did not interact with any other thioredoxin fusion except GCN4 (not shown).

All λ cI -LTTR proteins interacted with their respective thioredoxin fusion, as indicated by the black boxes in Figure 2. Surprisingly, some heterotypic interactions were observed, particularly with the Trx-HdfR fusion against λ cI -YafC, λ cI -CynR and λ cI -YiaU, as indicated by the gray boxes.

### Expansion of the Grid

We next tested the λ cI-LTTR fusions against 20 other Trx-LTTR fusions. The resultant interactions are shown in Figure 2. Several Trx-LTTR fusions had numerous interactions with the λ cI-LTTR fusions, including Trx-Cbl, -OxyR, -PerR and -YbeF. Others, such as LysR, NhaR and YneJ showed only one or two interactions. Many showed no interactions with the λ cI-LTTR fusions tested.

### Results of Both Grids

Of the 216 potential heterotypic interactions, 37 were detected, which allowed the LTTRs to be divided into 3 classes: I) those whose Trx-LTTRs did not interact with any others, II) LTTRs whose Trx fusions formed only one or two interactions (CynR, LysR, NhaR, YneJ and YiaU), and III) LTTRs whose Trx fusions were promiscuous in their interactions with other LTTRs (Cbl, OxyR, PerR, YbeF and YdaK). Note, however, that all eight cI fusions interact with at least three noncognate Trx fusions.

### Phylogenetic analysis

We used the phylogenetic tree from PFAM for the LysR substrate domain (PF03466) to examine the evolutionary relationships between the LTTRs used in this study. The average pairwise branch lengths separating all pairs of *E. coli* LTTRs, members of the tested set, and the interacting pairs were 2.51±0.45, 2.52±0.40, and 2.41±.43, respectively.

## Discussion

Coupling the assembly of LTTRs to the DNA binding activity of phage λ repressor allowed us to use a single assay based on negative dominance to perform pair-wise testing of 216 potential interactions among LTTRs that normally recognize different DNA sequences.

With the caveats discussed below, the overall pattern we observed is consistent with the idea that evolution selects for diversification of assembly specificity within families of paralogous proteins. Although most LTTRs (class I) have few detectable interactions with the other tested LTTRs, cross-interactions are observed and none of the cI-LTTR fusions was resistant to all of the noncognate Trx-LTTR fusions. Since we could not test all combinations, we cannot tell whether the LTTRs whose Trx fusions were in class I interact with other noncognate LTTRs.

In principle, noncognate negative dominance could be due to either specific or nonspecific protein-protein interactions. Nonspecific interactions could affect only a subset of partners if different cI-LTTRs vary in their sensitivity to such nonspecific effects. For example, LTTRs with intrinsically weaker multimerization would be more sensitive to a nonspecific competitor. We would expect that such nonspecific mechanisms would show a hierarchy where, although different cI fusions would react with different subsets of the Trx fusions, the fusions could be ranked in terms of both the ability of the Trx fusions to elicit the nonspecific effect, and the sensitivity of the cI fusions to the effect. The patterns of inhibition seen with the class II and class III Trx-LTTRs are inconsistent with a single nonspecific mechanism of this kind.

The class III Trx-LTTR fusions collectively interact with all eight of the cI fusions, but there seems to be no obvious pattern for which cI fusions are sensitive to the overexpression of each class III Trx fusion. Within the subset of possible interactions we could test, the pattern of cross-interaction also does not seem to fall into disjoint clusters, as we might expect if specific interactions behaved as stable traits over the evolution of the LTTR family.

This lack of pattern suggests that the cross-interaction among LTTRs may reflect independent evolution of interactions, which need not even involve the same interface residues. Consistent with this view, phylogenetic analysis of interacting and noninteracting pairs showed no evidence that the interactions we observed are generally correlated with evolutionary distance. Although half of the eight cI fusions did interact with their closest relative, all of them interacted with LTTRs that are more distantly related than proteins with which they failed to interact in our assay. This is not surprising for the highly divergent paralogs we tested with pairwise sequence identies ranging from 5.45% (yiaU vs cysB) to 33.1% (perR vs gcvA).

Previous systematic studies of oligomerization in OxyR and CynR, showed that although the surfaces involved in homodimerization of their regulatory domains are superficially similar, distinct residues and residue interactions are important for oligomerization of these LTTRs [21], [22]. Cross-interactions could thus reflect the plasticity of subunit interfaces, allowing evolution to find different combinations of interactions to build similar quaternary structures.

The physiological significance of the cross-interactions is unclear. It is formally possible that heteromultimeric LTTRs form functional transcription factors with different physiological roles from the homomultimers. However, we do not think this is likely in most cases of cross-interaction. Formation of heteromultimers could interfere with the normal function of LTTRs, just as it interferes with the λ repressor fusions. However, it is likely that some heteromultimerization can be tolerated. Our assay cannot determine the relative affinities of LTTRs homomultimers vs. heteromultimers, so it is possible that homomultimers are favored and heteromultimers are only observable under our artificially high overexpression of the Trx fusions. But even if homomultimers and heteromultimers form with equal affinity, note the concentration of inhibitors *in vivo* may be inadequate to have a significant inhibitory effect. Transcription factors are often expressed at low steady state levels, of the thirty-eight LTTRs we examined, only two had detectable peptides in a mass-spectrometry based catalog of protein abundance in *E. coli* [23].

As with all fusion-based systems, ours has limitations and is likely to have false positives and false negatives. Negative dominance occurs only if the inhibitory heteromultimerization can drive the concentration of an active homomultimeric cI fusion below the threshold needed to to block phage infection. Empirically, we find that this requires a large excess of the inhibitory Trx fusion to drive the equilibrium depicted in Figure 1 far enough to the right. However, the expression system used for the Trx-LTTR fusions could be so far above physiological concentrations of the LTTR that interactions that are not biologically relevant might be detected; the physiological levels of each native LTTR has not been determined. However, differences in the expression of the Trx-LTTRs is unlikely to account for the differences in promiscuity of the observed interactions. The fusions are C-terminal fusions that use the same transcription and translation signals for their expression. In the cases where we have examined accumulation of the fusion proteins after induction, the proteins accumulated to similar levels (data not shown).

## Methods

### Construction of Fusions

#### cI-LTTR fusions

Plasmid-borne lambda cI-LTTR fusions that confer immunity to infection by phage lambda (left column of grid in Figure 2) were previously identified in a screen for homotypic interactions in *E. coli* K-12 [20]. Each of these fusion proteins contains the DNA-binding domain of λ cI Repressor fused to a full-length (or almost full-length) LTTR protein. These are expressed from a plasmid vector that carries ampicillin resistance and has the M13 single-strand replication origin [24].

#### Trx-LTTR fusions

Plasmid-borne Trx-LTTR fusions were constructed using the Gateway (Invitrogen) system. For cynR, gcvA, iciA, ilvY, pssR, yafC, yiaU, and ynfL, the cI fusions described above were used as PCR template DNA. These LTTR genes were amplified using Pfx Platinum DNA polymerase (Invitrogen) and primers *att*B1 and *att*B2 (PAGE purified, IDT, Iowa), which attach Gateway cloning sites. The sequences of the primers were: *attB1*: 5′ GGG GAC AAG TTT GTA CAA AAA AGC AGG CTA CAA GGA CGA CGA TGA CAA G 3′; *attB2*: 5′ GGG GAC CAC TTT GTA CAA GAA AGC TGG GTC TTT CGG GCT TTG TTA GCA G 3′. The sizes of the amplified products were verified by agarose (1% in TBE) gel electophoresis.

The other ORFs were obtained as plasmids from plasmids obtained from H. Mori (Keio University) in the Gateway destination vector pAZ20. pAZ20 is a modified version of pLM1000 [25] in which the amber stop codon between the λcI DNA-binding domain and the *attL* Gateway site was removed and a *Sfi*I site was created between the Gateway sites *via* site-directed mutagenesis. After purification using the Qiagen PCR Clean-up Kit, each PCR product was recombined into pDONR201 (Invitrogen) using the Gateway BP reaction and transformed into either E. coli K-12 MC1061 [26] or Mach T1 (Invitrogen).

Trx-LTTR fusion plasmids were constructed by Gateway attL-attR reaction between these entry clones and plasmid pJM198 (this study). pJM198 is derived from pBAD-DEST49 (Invitrogen), where the origin of replication and ampicillin resistance gene are replaced by the origin and tetracycline resistance gene from pACYC184.

### Negative-Dominant Assay

We modified the negative-dominance assay based on the λ cI repressor system developed by Zeng *et al.* [19] to detect protein-protein interactions. As illustrated in Fig. 1, the wild-type DNA-binding domain of l cI is fused to an LTTR protein that can oligomerize the DNA-binding domain and confer immunity to infection by λ, even when the cI^+^-LTTR fusion protein is transcribed from a weak, constitutively expressed promoter. When a second construct encoding the same LTTR protein now fused to thioredoxin (Trx) is introduced to the cells and the fusions protein expressed at high levels, the concentration of cI^+^-LTTR homooligomers is decreased by the formation of cI^+^-LTTR/Trx-LTTR heterooligomers and the cells become sensitive to λ infection. The ability of different LTTR proteins to form heteroligomers *in vivo* was tested using pairwise combinations of cI^+^ and Trx fusion proteins.

### Construction of Double Strains

Strains containing the two plasmid constructs to be tested were constructed using M13 transduction. Bacteriophage M13 RV-1 was used to package the plasmids carrying the immune λ cI-LTTR fusions. Lysates were made on strains growing in 2XYT plus ampicillin (200 µg/mL). Recipient strains containing the Trx-LTTR fusions were grown overnight in 2XYT plus tetracycline (20 µg/mL). Approximately 50 µl of the recipient cultures was transferred to the wells of a sterile 96-well plate followed by the addition of 5 µL of the appropriate M13 lysate. After incubation for 10 min at room temperature, 100 µL of LB was added to each well, and the plate was covered with Airpore tape (Qiagen). After 2 h incubation at 37°C, cells containing both plasmids were selected by using a 96-prong frogger to transfer cells to LB plates containing ampicillin and tetracycline. After overnight incubation at 37°C, the entire grid was stamped onto fresh LB-ampicillin-tetracycline plates and once again incubated overnight at 37°C. Cells were taken from these spots and tested for immunity by cross-streaking as described below.

### Cross-streak Assays

Cross-streak assays were performed to assay the ability of a Trx-LTTR fusion protein and a cI-LTTR fusion protein to form heterooligomers. Briefly, parallel lines of phage λKH54 and λi^21c^ were drawn on plates containing tryptone broth (TB) medium, TB +0.2% glucose, or TB +0.2% arabinose and allowed to soak into the plate. Colonies of strains to be tested were dragged across the lines of phage. Plates were incubated at 37°C for six hours and the phenotype noted. Fusion proteins that can form heterotypic interactions will be sensitive to λ if the cI^+^ homooligomer levels fall below the critical level required for immunity to λKH54 and thus have a sensitive phenotype. Immunity to λ is scored when the cI^+^/cI^+^ complex is more stable than a cI^+^/cI^−^ complex.

### Phylogenetic analysis

A phylogenetic tree built from the LysR substrate domain (PF03466) of 33481 entries was downloaded from PFAM (http://pfam.sanger.ac.uk/family/PF03466#tabview=tab4) [27] as a Newick formatted text file. A BioPerl script was used to extract pairwise distances between nodes for all of the *E. coli* LTTRs. Distributions of these distances were analyzed using Microsoft Excel. Pairwise amino acid identity was calculated using multiple sequence alignment with T-Coffee [28].
